# Isolation, Identification and Activities of Natural Antioxidants from *Callicarpa kwangtungensis* Chun

**DOI:** 10.1371/journal.pone.0093000

**Published:** 2014-03-25

**Authors:** Hao Cai, Zhiyong Xie, Guanghui Liu, Xiuman Sun, Guangtian Peng, Baoqin Lin, Qiongfeng Liao

**Affiliations:** 1 School of Chinese Materia Medica, Guangzhou University of Chinese Medicine, Guangzhou, China; 2 School of Pharmaceutical Sciences, Sun Yat-sen University, Guangzhou, China; University of Sassari, Italy

## Abstract

Reactive oxygen species leads to some diseases associated with oxidative stress. *Callicarpa kwangtungensis* Chun (CK) is a common remedy in traditional Chinese medicine and possesses diverse biological activities involving antioxidant properties; its main compounds phenylethanoid glycosides (PG) and flavonoids are always reported as antioxidants. In order to develop CK as a safe and activated antioxidant, our investigation was performed to validate antioxidant properties and assess which types of compounds (similar polarity or similar structure), even which compounds, played the role of antioxidants. The extracted compounds of CK were analyzed qualitatively and quantitatively by HPLC-DAD-ESI-Trap MS and UV for their contents and antioxidant activities. The correlations between antioxidant activities and known contents were respectively counted and a semi-quantitative experiment was designed to screen antioxidant compounds of CK with HPLC-UV. The n-butanol fraction (BF) showed the highest total phenolic and flavonoid contents (TPC, TFC), and three PG (forsythiaside B, poliumoside and acteoside) contents. BF showed the significantly best (*P*<0.05) activities in most assays. There were significant correlations (*P*<0.05) between DPPH•, ABTS^+^•, •O_2_^−^ scavenging, Cu^2+^-chelating, anti-lipidperoxidation activities and TPC. BF also has significant antioxidant activities on CCl_4_-induced acute liver injury Mice and TBHP-reduced HepG2 cells. Nine PG (forsythiaside B, poliumoside, acteoside, alyssonoside, brandioside and their derivatives) and one flavone (rhamnazin) were screened out as antioxidants. BF in CK contained abundant polyphenolic, which reflected some definite antioxidant properties. The antioxidant compounds consisted at the least of nine PG and one flavone.

## Introduction

Although oxygen is critically important for most of the organisms on earth, it may be toxic and mutagenic for living organisms. In normal metabolism of living organisms, reactive oxygen species (ROS) are generated inevitably. ROS play a useful role in signal transduction, but excessive production of ROS leads to oxidative stress damage to cellular structures, such as lipids, DNA and proteins, which is a result of imbalance between the antioxidant system and the formation of ROS in vivo [1]. As a result, oxidative stress is clearly associated with the etiology of a wide range of chronic and acute disease such as malignant tumors, inflammation, cataracts, Parkinson's and Alzheimer's disease, hypertension, diabetes, atherosclerosis, cardiovascular diseases, cell death, and some immune disorders and the aging process [1], [2]. An antioxidant is “any substance that, when present at low concentrations compared to those of an oxidizable substrate, significantly delays or prevents oxidation of that substrate” [3]. They have the aptitude to scavenge ROS by donating hydrogen atom or electron, chelating metal catalysts, activating antioxidant enzymes and inhibiting oxidizes. To protect the body from such effects, in addition to antioxidant enzymatic system, there are non-enzymatic biomolecules and proteins in living organisms, which act as antioxidant and free radical scavengers. But most of time, it is not enough to rely on the endogenous antioxidant to protect the body from such effects, so it is often recommended that we increase our intake of dietary antioxidants through our food. Natural antioxidants, particularly polyphenolic, especially from medical plants, are safer and also more bioactive compared with synthetic antioxidants [4]. Many researchers have focused on natural antioxidants, and numerous crude extracts and pure natural compounds have previously been reported to have antioxidant properties [4]. Therefore, the development and utilization of more effective antioxidants of natural origin are desirable.

*Callicarpa kwangtungensis* Chun (CK) is frequently used as a traditional Chinese medicine and its officinal parts are twig and leaf. The distribution of CK in China is at the altitude from 100 to 1000 metres in places such as Jiangxi, Guizhou, Guangdong Province etcetera. Modern medicine has indicated that it possesses various pharmacological effects involving antioxidant ability, hemostatic activity, analgesic activity, anti-inflammatory, and antimicrobial activity [2]. CK is also the major effective ingredient in a Chinese patent medicine called “Kang-Gong-Yan Tablet” which was used for the treatment of gynecological diseases. The extracts of other Callicarpa species, such as *C. kochiana*, *C. bodinieri*, *C. japonica*, *C. macrophylla*, *C. cathayana C. H.* Chang, *C. dichotoma*, *C. formosana* Rolfe and *C. rubella* have been investigated for antioxidant effect and proved to own the antioxidant activity [5]. As reported in our unique previous article, CK possesses an antioxidant effect which is determined by only one free radical elimination method [6]. CK contains various phenylethanoid glycosides (PG) and flavonoids such as forsythoside B, poliumoside, acteoside, all of which are polyphenolic compounds and mentioned as excellent antioxidants in previous articles [7]. Some researches about the contents of CK compounds have shown that three PG (forsythoside B, poliumoside and acteoside) are the major compounds in CK [2]; they are likely to be correlated with the antioxidant effect of CK.

Many natural antioxidants may act as pro-oxidant based on the dose and on the ambient redox conditions. Some main mechanisms about the common antioxidants acting as pro-oxidant have been described. For example [8]–[10], because of many conjugated double bonds, a large dose ofβ-carotene is susceptible to damage by ROS to promote oxidation. A large dose of vitamin E has been proved to lower the antioxidant enzyme activity by signaling pathways to reduce the antioxidant defensive systems and act as pro-oxidant. While α-lipoic acid scavenges ROS, the intermediates, such as HSRS·, have more strong oxidative activity compared with ROS.

In order to evaluate the potential antioxidant functions of bio factors in natural plant extracts as prophylactic agents or food additives, it is important to employ a number of analytical techniques since the antioxidant potency can differ substantially according to the physical and chemical parameters of the systems used for their characterization. Since the polyphenolic compounds, including flavonoids, contain a large amount of phenolic hydroxyl,they have a very strong effect on antioxidation and elimination of free radicals. For our investigation, the extract of CK was first separated according to different polar fraction, with the method for liquid-liquid extraction. Next,the compounds from different polar fraction were qualitatively and quantitatively analyzed,and contents of total phenolic and total flavonoid were assayed. Then,for the purpose of a detailed investigation of the in vitro antioxidant potentials of CK, seven methods representing different mechanisms of antioxidant protection of organisms from oxidative stress, namely, DPPH·scavenging, ABTS^+^·scavenging, ·OH scavenging, ·O_2_^−^ scavenging, Cu^2+^-chelating, Fe^3+^ reducing antioxidant power (FRAP), and anti-lipidperoxidation (anti-LP), were used to evaluate CK antioxidant activity. A mice study and a cell test were carried out to verify the antioxidant activity of BF. At last, the correlations between antioxidant activity from seven methods and known contents were counted respectively, and a semi-quantitative experiment was designed to screen antioxidant compounds of CK.

## Materials and Methods

### 2.1. Ethics Statement

In this study, no researches involving human participants were covered. All experimental protocols were approved by the Animal Management Rules of the Ministry of Health of the People's Republic of China (documentation Number 55, 2001, Ministry of Health of PR China). All surgery was performed under sodium pentobarbital anesthesia, and all efforts were made to minimize suffering. Because the plant materials were purchased from a GAP farming model base, no specific permits were required for the described field studies. The study was not privately owned or protected in anyway. The field studies did not involve endangered or protected species.

### 2.2. Chemicals and Reagents

Quercetin, gallic acid (GA), forsythiaside B, poliumoside, acteoside were purchased from National Institute for the Control of Pharmaceutical and Biological Products (Guangzhou, China). 2,2-Diphenylpicrylhydrazyl radical (DPPH•), 2,2′-azino-bis (3-ethyl-benzothiazoline-6-sulfonic) diammonium salt (ABTS^+^), butylated hydroxytoluene (BHT), ascorbic acid (AA), pyrogallol, Sodium citrate (SC), soybean lecithin, murexide (5,5′-nitrilodibarbituric acid monoammonium salt), sodium salicylate, thiobarbituric acid (TBA) and trichloroacetic acid (TCA) was purchased from Sigma-Aldrich Trading Co. (Shanghai,China); Folin-Ciocalteu's phenol reagent was purchased from Merck Co. (Darmstadt, Germany). The kits of Methane Dicarboxylic Aldehyde (MDA), (superoxide dismutase) SOD, protein concentration were purchased from Nanjing Jiancheng Bioengineering Institute (Nanjing, China). Dulbecco's Modified Eagle Media (DMEM) was purchased from Gibco BRL, Life Technologies (Gaithersburg, MD, USA). Thiazolyl Blue Tetrazolium Bromide(MTT), tert-Butyl hydroperoxide solution(TBHP) were purchased from Aladdin Trading Co.(Shanghai,China). Acetonitrile, methanol and water were of HPLC grade; all other chemicals used were of analytical grade.

### 2.3. Plant Materials

The aerial parts of *Callicarpa kwangtungensis* Chun at maturity (2012–09) were purchased from a provincial and municipal CK GAP farming model base named Xin-Nong Callicarpa Professional Cooperatives, Pingxiang, Jiangxi Province, China (northern latitude: 113°87′, east longitude: 27°13′). A voucher specimen (No. Liuyu217) was deposited in Medicinal Herbarium, Guangzhou University of Chinese Medicine (Guangzhou, China). The aerial parts of plant were shade dried at room temperature for two weeks, chopped and ground mechanically(22 000 rpm, 1 min) with herbs grinder to mesh size 1 mm.

### 2.4. Instrumentation

A Shimadzu series LC-20AT HPLC instrument (Kyoto, Japan) equipped with a quaternary pump (LC-20AT), a diode array detector (SPD-M20A), an auto sampler (SIL-20A), and a column compartment (CTO-20A); a Thermo LCQ FLEET series mass spectrometer with an electrospray interface (ESI) (CA, USA) used for qualitative analysis as HPLC-ESI-Trap MS, a TDL-40B high-speed centrifuge (Anting, Shanghai), a BP211D electronic balance (Sartourius, Germany), an XTY5107922 vortex-mixing mill (XT, Beijing), a UV-2000 spectrophotometer (Unico, Shanghai), an Infinite M1000 Mutil Reader (Tecan, Switzerland) and an LK-500 small-sized herbs grinder (NF, Guangdong), an RE-2000E rotatory evaporator (YH, Henan) equipped with a DXLS-080 Circulating water vacuum pump(XL, Zhejiang) were used.

### 2.5. Preparation of the Extracts

The dried powder (20 g) of CK was extracted with 1 L of 50% ethanol at 80°C for 60 min under reflux; the extraction was repeated for three times. The extracts were then combined, filtered, and concentrated under reduced pressure(0.098 MPa)with a rotatory evaporator; the yield of residue obtained was counted. Of the residue obtained, 3 g was suspended in water and successively partitioned with petroleum ether, ethyl acetate, and n-BuOH. Each fraction was concentrated under reduced pressure to yield a petroleum ether fraction (PEF), an ethyl acetate fraction (EAF), an n-butanol fraction (BF) and a remainder (aqueous) fraction (AF). All the fractions were stored at 4°C and dissolved in 50% ethanol or methanol prior to analysis.

### 2.6. Phytochemicals Characterization

#### 2.6.1. Total Phenolic Content Assay (TPC)

Total Phenolic Content was determined according to the Folin-Ciocalteu specterophotometric method described by Sharma [7]. Each sample (0.1 mL, with appropriate dilution if necessary) was added to 0.75 mL freshly diluted (10-fold) Folin-Ciocalteu reagent. The mixture was allowed to equilibrate for 5 min and then mixed with 0.75 mL of Na_2_CO_3_ aqueous solution (0.6 M). After incubation at room temperature for 90 min, the absorbance of the mixture was read at 725 nm. The standard curve was prepared using different concentrations (0.7–3.5 mg/mL) of pyrogallic acid and the results were expressed as mg pyrogallic acid equivalents (PAE) per gram of extract.

#### 2.6.2. Total Flavonoid Content Assay (TFC)

Total flavonoid content was measured using the NaNO_2_-Al(NO_3_)_3_ method from Sharma [7], with minor modifications. In brief, 0.1 mL of properly diluted sample was mixed with 0.3 mL NaNO_2_ aqueous solution (0.7 M). The mixture stood at room temperature for 6 min, followed by the addition of 0.3 mL Al(NO_3_)_3_ aqueous solution (0.9 M). After incubation at room temperature for 6 min, 1 mL NaOH aqueous solution (2.5 M) was added to the mixture. The absorbance was read at 508 nm on a spectrophotometer. The standard curve was prepared using different concentrations (0.05–0.25 mg/mL) of quercetin and the results were also expressed as mg quercetin equivalents (QE) per gram extract.

#### 2.6.3. HPLC-DAD-ESI-Trap MS system

The ME and its four fractions were respectively diluted with methanol to produce a final concentration of 5 mg/mL. Five samples were separated on a Phenomenex Luna C_18_ column (250 mm×4. 60 mm,5 μm). The mobile phase consisted of acetonitrile (A) and 0.1% acetic acid (v/v, B). The following gradient elution program was used: 15–20% A for 0–6 min, 20–20% A for 6–16 min, 20–25% A for 16–30 min, 25–50% A for 30–31 min, 50–50% A for 31–35 min, 50–65% A for 35–65 min, and 65–95% A for 65–68 min. The flow rate was 1.0 mL/min and the split ratio was 5∶1. The column temperature was set to 30°C. The UV detector was monitored at 254 nm.

The mass spectrometer was operated in negative ion mode with full scan mass spectra over the m/z range 50–1000, a capillary voltage of −20 V, a capillary temperature of 350°C, a spray voltage of 4 kV, a spray current of 0.1 μA, a sheath gas flow rate of 5.0 L/min, and a collision energy of 35.0 V. Thermo Xcalibur controlled all the operations, acquisition, and analysis of data.

#### 2.6.4. The Determination of Three Phenylethanoid Glycosides

HPLC analysis of five samples with same concentration (0.2 mg/mL) for three PG (forsythiaside B, poliumoside, acteoside) was carried out. The method for determination of three PG came from a previously published article [11]. The separation was performed on the same C_18_ column with a mobile phase of acetonitrile-0.1% acetic acid (20∶80). The samples (10 μl) were injected into the HPLC apparatus. The flow rate and the column temperature were identical with those of the qualitative analysis of CK. All the chromatograms were monitored at 330 nm. All samples were assayed in triplicate. The data were processed with Shimadzu 2.0 ChemStation software.

### 2.7. In Vitro Antioxidant Assays

#### 2.7.1. DPPH• Scavenging Activity Assay

The DPPH• scavenging activity was determined by the method of Liu [12], with a slight modification. The samples (0.2–1.2 mg/mL in 50% ethanol, 0.1 mL) were mixed with 3.0 mL of 0.1 mM DPPH that was dissolved in 95% ethanol. The mixture was then shaken vigorously using a mixer, and measured at 517 nm after incubation for 30 min at 30°C in the dark. BHT at 0.2–1.6 mg/mL was used as a positive control. The DPPH• scavenging activity was calculated using the following equation:
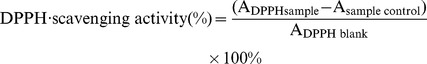



Where A_DPPH_ _sample_ is the value for 0.1 mL of sample solution mixed with 3.0 mL of DPPH solution; A _sample control_ is the value for 0.1 mL of sample solution mixed with 3.0 mL of 95% ethanol; and A_DPPH blank_ is the value for 0.1 mL of 50% ethanol mixed with 3.0 mL of DPPH solution.

#### 2.7.2. ABTS^+^• Scavenging Activity Assay

The ABTS^+^• scavenging activity was determined according to the method of Huang [13]with a slight modification. Aqueous solution of ABTS^+^ (7 mM) was oxidized with potassium peroxodisulfate (2.45 mM) for 16 h in the dark at room temperature. The ABTS^+^• solution was diluted with purified water to an absorbance of 0.75±0.05 at 734 nm. The samples (0.1-0.7 mg/mL in 50% ethanol, 0.1 mL) was mixed with 3.0 mL of diluted ABTS^+^• solution, the mixture was then shaken vigorously and the decrease of absorbance was measured at 734 nm after 6 min at 37°C in the dark. BHT at 0.06-0.6 mg/mL was used as a positive control. The ABTS^+^• scavenging activity was calculated using the following equation:
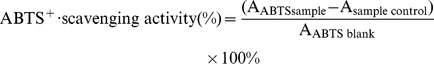



Where A_ABTS_ _sample_ is the value for 0.1 mL of sample solution mixed with 3.0 mL of _ABTS_ solution; A _sample control_ is the value for 0.1 mL of sample solution mixed with 3.0 mL of purified water; and A_ABTS blank_ is the value for 0.1 mL of 50% ethanol mixed with 3.0 mL of ABTS^+^• solution.

#### 2.7.3. •OH Scavenging Activity Assay

A modified version of Smirnoff and Cumbes [14]was used to measure •OH scavenging activities of samples. A mixture of 0.5 mL FeSO_4_ (2 mM) and 0.5 mL of sodium salicylate aqueous solution (0.2 mM) was mixed with 0.1 mL of the sample (0.25–4 mg/mL in 50% ethanol). Then, 0.5 mL H_2_O_2_ (0.16 mM) was added, which started the •OH scavenging reaction. The mixture was incubated at 37°C for 30 min, and the absorbance was measured at 510 nm. AA at 0.25–4 mg/mL was used as a positive control. The capability of scavenging the •OH was calculated, using the following equation:
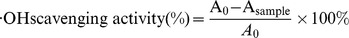



Where A_0_ represents the absorbance of the mixture in which the sample was replaced with 50% ethanol;A_sample_ represents the absorbance of the sample mixture (the sample absorbance at this wavelength is deducted).

#### 2.7.4. •O_2_^−^ Scavenging Activity Assay

The •O_2_^−^ scavenging activity was assessed using the inhibition of pyrogallol autoxidation method [14]. The samples (0.25–4 mg/mL in 50% ethanol, 0.1 mL) was mixed with 1.5 mL Tris-HCl buffer (0.1 M, pH 7.4) containing EDTA (1 mM). When 0.1 mL pyrogallol (6 mM) was added, the mixture was shaken at room temperature. Then the absorbance at 325 nm of the mixture was measured against the Tris-HCl buffer as blank every 30 s for 3 min, immediately. AA at 0.03–4 mg/mL was used as a positive control. The slope of the correlation of absorbance with time was calculated. The •O_2_^−^scavenging ability was calculated as:
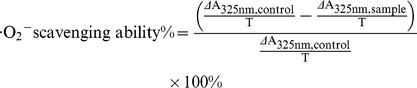



Here, ΔA_325nm, control_ is the increase in A_325nm_ in the mixture without the sample, and ΔA_325nm, sample_ is the increase in A_325nm_ in the mixture with the sample; T = 3 min.

#### 2.7.5. Cu^2+^-Chelating Activity Assay

The Cu^2+^-chelating activities were measured by a complexometric method of Li [15], with a slight modification. Briefly, 3.0 mL murexide solution (hexamine-HCl buffer containing 30 mM KCl, 0.25 mM, pH 5.3) was added to CuSO_4_ aqueous solution (0.1 mL, 2 mM).After incubation for 1 min at room temperature, 0.1 mL of the samples (0.2–2.5 mg/mL in 50% ethanol) was added. Then, the mixture was shaken vigorously and left at room temperature for 10 min. The absorbance was measured at 485 nm and 520 nm. The absorbance ratio (A_485_/A_520_) reflected the free Cu^2+^ content. SC at 0.08–2.3 mg/mL was used as the positive control. The relative Cu^2+^-chelating activity was calculated using the following equation:
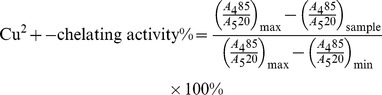



Where 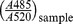
is the absorbance ratio of the mixtures with samples, while
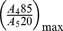
is the absorbance ratio of the mixture without samples, and
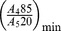
is the absorbance ratio of the mixtures without CuSO_4_ aqueous solution and sample in the test.

#### 2.7.6. Fe^3+^ Reducing Antioxidant Power (FRAP)

The reducing power of all samples was determined by a method described by Li [16], using a modified Fe^3+^to Fe^2+^ reduction assay, whereby the yellow color of the test solution changes to various shades of green and blue, depending on the reducing power of the samples. In brief, 0.1 mL of the samples (0.2–1.8 mg/mL in 50% ethanol) was mixed with 0.4 mL Na_2_HPO_4_/KH_2_PO_4_ buffer (pH 6.64) and 0.25 mL K_3_Fe(CN)_6_ aqueous solution (3.0 mM). After the mixture was incubated at 50°C for 20 min, 0.25 mL TCA aqueous solution (0.6 M) was added and the resultant mixture centrifuged at 480 g for 10 min. The supernatant (0.75 mL) was mixed with the same volume of FeCl_3_ aqueous solution (3.7 mM). After standing for 10 minutes, absorbance of the mixture was stable and read at 700 nm. BHT at 0.3–1.8 mg/mL was used as positive control. The relative reducing ability of the sample was calculated by using the formula:
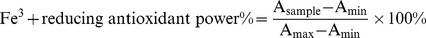



Where A_max_ is the absorbance of mixture in which K_3_Fe(CN)_6_ aqueous solution was replaced with an equal molar concentration of K_4_Fe(CN)_6_ aqueous solution and the samples were replaced with 50% ethanol; A_min_ is the absorbance without samples; A_sample_ is the absorbance of sample mixture (the sample absorbance at this wavelength is deducted).

#### 2.7.7 Antioxidant Effect on Lipidperoxidation (LP)

The antioxidant activity of the extracts was evaluated by the ability of different concentrations of plant extracts to inhibit LP in liposomes, induced by FeCl_3_/ascorbate system. This anti-LP assay was based on the method [17], with some modifications.

Lecithin was suspended in phosphate buffer (0.2 mM, pH 7.4) to obtain lecithin suspension (0.03 mM). This suspension was sonicated with a rod using an ultrasonic homogenizer at 30 s intervals for 10 min until an opalescent suspension was obtained.

The 0.2 mL lecithin suspension was preincubated with 0.2 mL of samples (1.6–8.7 mg/mL in 50% ethanol) or reference antioxidant (1.7–8.5 mg/mL GA) in the presence of 0.2 mL FeCl_3_ (1.2 mM). LP was initiated by adding 0.2 mL AA (1.2 mM), and the mixture was then incubated for 1 h at 37°C. It was added with 0.5 mL TCA aqueous solution (0.6 M) to stop the reaction, and centrifuged at 480 for 10 min. The supernatant (0.5 mL) was mixed with 1.0 Ml TBA (0.07 mM). The mixture was heated at 98°C for 20 min in a water bath. The formed (TBA)_2_-MDA (malondialdehyde) chromogen was measured at 532 nm to measure the antioxidant activity on LP. The relative antioxidant activity of the sample on LP was calculated by using the formula:
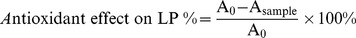



Where A_0_ represents the absorbance of the mixture in which sample was replaced with 50% ethanol; A_sample_ represents the absorbance of sample mixture (the sample absorbance at this wavelength is deducted).

#### 2.7.8. Antioxidant Activity of BF on CCl_4_-induced acute liver injury mice

Male KM mice (body weight: 18–22 g) were provided by Animal Department of Guangzhou University of Chinese Medicine (Guangzhou, China). They were housed in standard cages at a constant temperature of 22 °C ± 1 °C, relative humidity 55% ± 5% with 12 h light-dark cycle (08:00 to 20:00) for at least 1 week before experimentation began. The mice except for the control group were given CCl_4_ solution (10 mL/kg, 5% v/v) intraperitoneally in order to produce a CCl_4_-induced acute liver injury model, control mice received a comparable volume of olive oil (i.p).

For the liver protection experiments [18], control and CCl_4_-treated mice were orally administered, and distilled water was used as the non-therapeutic control. The positive control group was given Berberine (40 mg/kg) orally for 5 consecutive days. The BF groups of mice were orally administered BF (100, 300 and 900 mg/kg) for 5 consecutive days. One hour after the last administration of the experimental drugs, a CCl_4_-induced acute liver injury model was produced. Twenty-four hours after CCl_4_ injection, the mice were sacrificed under anesthesia and liver tissue was collected to allow the protein content, SOD and MDA to be measured.

The 10% solution of tissue homogenate was prepared as follows. Pieces of liver were homogenized in 9 volumes (w/v) ice cold saline and centrifuged at 3000 rpm for 10 min. The supernatants were separated and stored at −20°C for analysis. The protein content, SOD and MDA activities in liver tissue were determined using the respective commercial kits. Protein content of tissue homogenate was determined according to the Coomassie brilliant blue (CBB) colorimetric method, bovine serum albumin was used as standard. SOD activity was determined by the hydroxylamine method and expressed as units/mg protein, MDA activity was determined by the thibabituric acid method and expressed as nmol/mg protein.

#### 2.7.9 Determination of Cytotoxicity/Protective Effect of HepG2 cells

HepG2 human hepatoma cell line was obtained from Guangzhou University of Chinese Medicine (Guangzhou, China).HepG2 cells were cultured in dulbecco's modified eagle medium (DMEM), supplemented with 10% foetal bovine serum (FBS), HEPES buffer pH 7.4 (0.015 M), penicillin (0.134 mg/mL), streptomycin (1 mg/mL). Cells were incubated in a humidified incubator containing 5% CO_2_ and 95% air at 37°C and subcultured by trypsinisation when they reached confluence, and treated as described below [19].

Cells were plated in a 96-well plate (3.0×10^4^ cells per well) and incubated in a humidified incubator containing 5% CO_2_ and 95% air, at 37°C, for 24 h. Then, the cells were treated with various concentrations of BF (0.04–25 mg/mL) and incubated for 24 h. The culture medium was removed and replaced with a fresh medium. Cells were incubated with 0.5 mg/mL MTT for an additional 4 h. Subsequently, the cells were centrifuged at 2000 rpm, at 4°C, for 5 min. The culture medium was discarded and the purple formazan crystals were dissolved with DMSO (100 μL/well). Absorbance was monitored at 590 nm using a microplate reader. The result was expressed as percentage of viable cells compared to the control group (cell only).

Cells were plated in a 96-well plate (3.0×10^4^ cells per well) and incubated in a humidified incubator containing 5% CO_2_ and 95% air, at 37°C, for 24 h. Then, cells were pretreated with various concentrations of BF (0.04–5 mg/mL) and incubated for 24 h. The culture medium was removed and replaced with a fresh medium containing 0.5 mM TBHP and further incubated for 2 h. Subsequently, cell viability was determined using MTT dye solution as described above. The result was expressed as a percent-age of viable cells compared to the control group (cell+0.5 mM TBHP), while VC at 2.5 μg/mL was used as a positive control.

### 2.8. Rapid Screening of Antioxidants from CK by HPLC-UV

0.1 mL ME (5 mg/mL in 50% ethanol) was mixed with 0.9 mL of 5 M DPPH that was dissolved in 95% ethanol. The mixture was then shaken vigorously using a mixer, then the mixer filtered with 0.2 μm millipore filter. Using the method of qualitative analysis of CK, the mixture was assayed in triplicate with HPLC -UV. The mixture in which the same volume of 95% ethanol replaced 5 M DPPH solution was used as a blank control. Comparing the peak area of every identified compound in two chromatograms, some identified compounds can be rapidly screened as antioxidants. After the antioxidants reacted with DPPH•, the amount of decreasing peak area depended on binding affinities. The stronger the antioxidants, the more binding the affinities, and the more the decrease in peak area. So, The relative binding affinities (RBA) defining the antioxidant activity was calculated by using the formula:
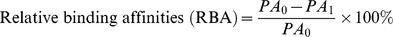
where PA_0_ represents the peak area of blank control; PA_1_ represents the the peak area of mixer of ME and DPPH•.

### 2.9. Statistical Analysis

Statistical analysis data were given as the mean ± SD of three measurements. Some values were calculated by linear regression analysis. Significant differences were performed using the T-test. Correlations were expressed as Pearson correlation coefficients. The analysis was performed using SPSS software (v.12, SPSS, USA).

## Results and Discussion

### 3.1. Phytochemical Characterization

Due to the complicated constituents and pharmacological diversities of plant materials, in vitro bioassay-guided fractionation has been effectively applied to screen the biological activities that provide important indications for investigating the characteristics of active components [20]. ME and its four fractions obtained from CK were fractionated through solvent-solvent partitioning to obtain four fractions of PEF, EAF, BF, and AF. As shown in Table 1, the yield extraction of ME was 19.79%,and the yield extraction of its four fractions were 0.81%, 1.64%, 6.69%, and 11.15%, respectively. Data from the yield extraction showed that more than half of the extract existed in AF.

**Table 1:** TPC and TFC in different extracts of CK.

Sample	% yield extraction	TPC as mg Pyrogallic acid equivalents (PAE mg/g extrac)	TFC as mg quercetin equivalents (QE mg/g extrac)
			
**ME**	19.79±0.47^e^	84.61±3.70^c^	25.18±0.92^c^
**PEF**	0.81±0.02^a^	7.36±0.26^a^	ND
**EAF**	1.64±0.67^b^	107.00±2.9^d^	10.58±0.31^a^
**BF**	6.69±0.21^c^	152.10±3.4^e^	37.27±1.30^d^
**AF**	11.15±0.42^d^	43.02±2.0^b^	17.24±0.74^b^

Each value in the table is represented as Mean ± SD (n = 3) Means not sharing the same letter are significantly different (LSD) at P<0.05 probability level in each column.

#### 3.1.1 Total Phenolic and Flavonoid Contents

In the present study, TPC of ME and its four fractions were determined using the Folin-Ciocalteu method and expressed as milligram Pyrogallic acid equivalents per gram extract (PAE/g), shown in Table 1. The results varied from 7.36±0.26 mg PAE/g extract (PEF) to 152.1±3.40 PAE/g extract (BF). In the aspects of TPC,BF was higher than EAF (P<0.05) and they showed significantly high (P<0.05) TPC compared to the other fraction and ME. Phenolic compounds such as flavonoids, phenolic acids, and tannins were considered to be major contributors to the antioxidant activity of plants. TFC were determined by NaNO_2_-Al (NO_3_) _3_-NaOH colorimetry and expressed as mg quercetin equivalents (QE)/g extract. The results, shown in Table 1, varied from 0 mg QE/g extract (PEF) to 37.27±1.30 mg QE/g extract (BF).The order of TFC was similar to that of TPC and any fraction from CK extract was significantly different with another fraction. It was worth noting that TFC can't be detected in PEF, because of the polar of flavonoid compounds. In the same fraction, the TPC was more than double the TFC; this phenomenon was normal, because almost all the flavonoid components were regarded as polyphenols.

Phenolic compounds possess the ideal chemistry for antioxidant activity because they have high reactivity as hydrogen or electron donors and are also capable of chelating metal ions [21]. These antioxidants also possess diverse biological activities, such as anti-inflammatory, anti-atherosclerotic, and anti-carcinogenic activities. These activities may be related to their antioxidant activity [22]. Thus, TPC and TFC of CK were also evaluated. Polyphenolic flavonoids occurr ubiquitously in food and medicinal plants; they occur as glycosides and contain several phenolic hydroxyl groups. They are known for their efficient radical scavenging activity owing to their hydroxyl group at various positions and an ortho-dihydroxy structure in their B ring [23]. Recent investigation has shown that many flavonoids and related polyphenols contribute significantly to the antioxidant activity of many fruits, vegetables, and medicinal plants [24].

A research [6] on the determination of TPC and TFC from seven Callicarpa species has indicated that TPC of aqueous extract of CK from Guangdong, China, was 0.97±0.07% and TFC was 1.32±0.02%, which are significantly less than the TPC and TFC we have obtained. The huge difference may result from the factors of extraction solvent and the origin of the medicines. The TPC and TFC of seven Callicarpa species ranged from 0.97±0.07% to 2.93±0.01%,and from 1.05±0.06% to 3.75±0.11%, respectively.

#### 3.1.2. HPLC-DAD-ESI-Trap MS for Qualitative Analysis

The chemical constituents of CK were characterized by the developed HPLC-DAD-ESI-Trap MS method. Most target compounds showed higher response in negative ion mode than positive mode, and can be characterized by the MS/MS fragments in negative ion mode. Therefore, negative ion mode was employed in this study, while positive mode was employed when necessary to provide complementary information.

**Figure 1:**
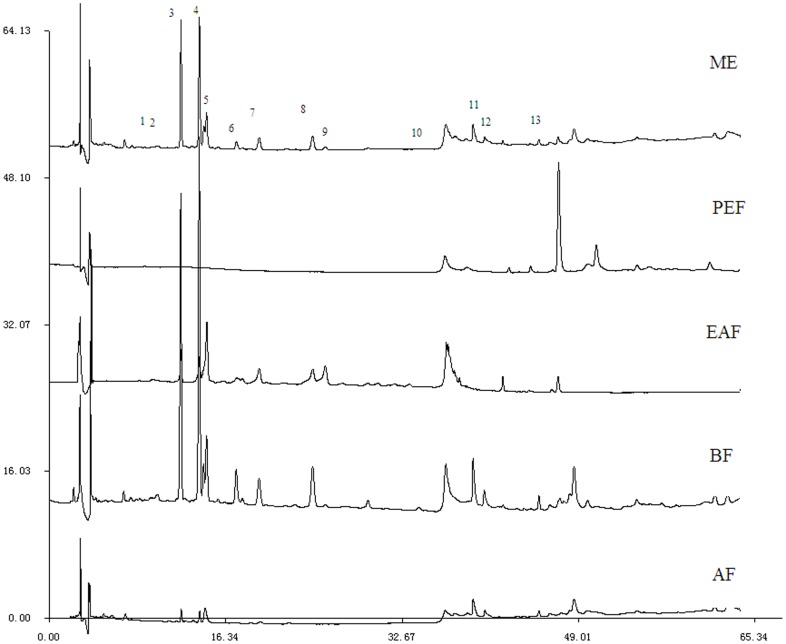
HPLC chromatograms of ME, PEF, EAF, BF, AF of CK detected at 254 nm.

**Figure 2:**
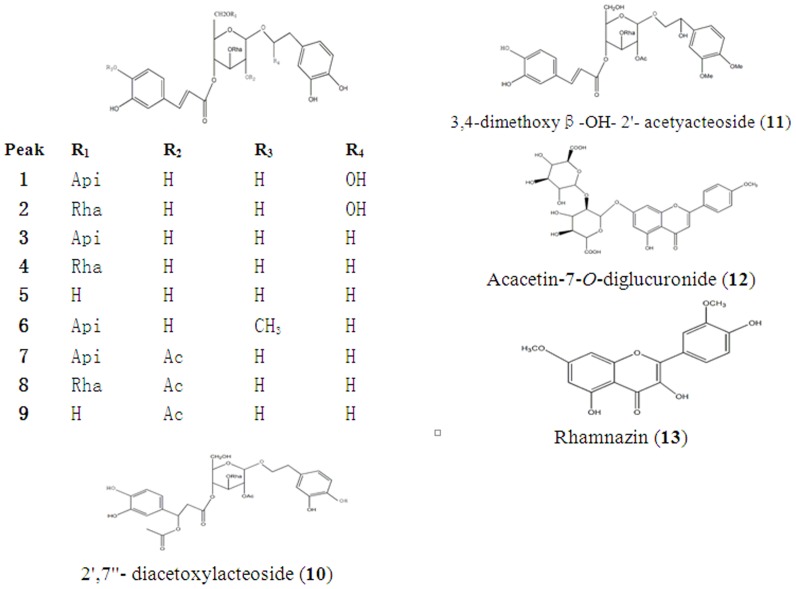
Chemical structures of 13 compounds identified from CK (Api, apiosyl; Rha, rhamnosyl; Ac, acetyl moiety).

The molecular weight of the compounds was determined by the predominant [M−H]^−^ in full-scan mass spectra. Since most of chemical constituents of CK were characterized as a type of PG, the chemical constituents can produce some characteristic fragments with the specific collision energy, such as caffeoyl (162 u), acetyl moiety (42 u), rhamnose (146 u), apiose(132 u), H_2_O (18 u). Eleven PG and two flavones were identified from CK. Some compounds can be characterized according to literature [25]. Figure 1 indicates the compounds that existed in every extractive fraction directly. All 13 compounds (Figure 2, Tables 2) appeared in the chromatogram before the ratio of mobile phase reached 55∶45 (A∶B). Their contents in BF were the highest in four-fraction chromatogram and there were few identified compounds in PEF and AF.

**Table 2:** Identification of 13 compounds from CK by developed HPLC–DAD–ESI-Trap MS method.

NO.	T_R_(min)	[M-H]^−^(M/Z)	MS/MS fragments(M/Z)	Identification
**1**	7.5	771	753 [M-H-H_2_O]^−^	β-OH-forsythoside B
			591 [M-H-H_2_O-caffeoyl]^−^	
**2**	9.55	785	767 [M-H-H_2_O]^−^	β-OH-poliumoside
			605 [M-H-H_2_O-caffeoyl]^−^	
**3**	12.16	755	593 [M-H-caffeoyl]^-^	Forsythoside B
			461 [M-H-caffeoyl-Api]^−^	
			447 [M-H-caffeoyl-Rha]^−^	
**4**	13.87	769	607 [M-H-caffeoyl]^-^	Poliumoside
			461 [M-H-caffeoyl-Rha]^−^	
**5**	14.56	623	461 [M-H-caffeoyl]^−^	Acteoside
			135 [caffeoyl]-	
**6**	17.3	769	593 [M-H-caffeoyl]^−^	Alyssonoside
			447 [M-H-caffeoyl-Rha]^−^	
**7**	19.42	797	635 [M-H-caffeoyl]^−^	2′-forsythoside B
			593 [M-H-caffeoyl-CH_3_CO]^−^	
**8**	24.38	811	649 [M-H-caffeoyl]^−^	Brandioside
			607 [M-H-caffeoyl-CH_3_CO]^−^	
			461[M-H-caffeoyl-CH_3_CO-Rha]^−^	
**9**	25.53	665	623 [M-H-CH_3_CO]^−^	2′-acetylacteoside
			503 [M-H-caffeoyl]^−^	
			461 [M-H-CH_3_CO-caffeoyl]-	
			315[M-H-CH_3_CO-caffeoyl-Rha]^−^	
**10**	34.2	725	665 [M-H-CH_3_CO-H_2_O]^−^	2′,7″- diacetoxylacteoside
			503 [M-H-CH_3_CO-H_2_O -caffeoyl]^−^	
			461 [M-H-2CH_3_CO-H_2_O -caffeoyl]^−^	
**11**	39.23	711	651 [M-H-CH_3_CO-H_2_O]^−^	3,4-dimethoxy-β-OH-2′-acetyacteoside
			489 [M-H-CH_3_CO-H_2_O -caffeoyl]^−^	
			447 [M-H-2CH_3_CO-H_2_O -caffeoyl]^−^	
**12**	42.15	635	351 [2GlcA–H–H_2_O]^−^	Acacetin-7-O-diglucuronide
			289 [M-H-348]^−^	
			193 [GlcA]^−^	
**13**	45.33	329	314[M-H-CH_3_]^−^	Rhamnazin
			299 [M-H-CH_3_-CH_3_]^−^	

#### 3.1.3. The Determination of Three Phenylethanoid Glycosides

Three PG were determined in ME and its different fractions; the data were reported in Table 3. After ME was successively partitioned with PEF, EAF, BF, and AF, three PG focused on certain fractions. Forsythiaside B existed in BF absolutely and could not be determined in other fractions. The distribution of poliumoside was similar to that of forsythiaside B; about 96.27% of poliumoside existed in BF. The distribution of acteoside was more scattered than forsythiaside B and poliumoside, but the BF was the main fraction in which it was distributed (about 77.82% of acteoside), while about 12.64% and 9.54% of acteoside existed in AF and EAF.

**Table 3:** Three PG contents in different extracts of CK.

Sample	Forsythiaside B compound concentration(mg/g extract)	Poliumoside compound concentration(mg/g extract)	Acteoside compound concentration(mg/g extract)
**ME**	2.19±0.11	3.43±0.13	0.98±0.04
**PEF**	ND	ND	ND
**EAF**	ND	0.35±0.01	1.2±0.03
**BF**	7.30±0.25	11.21±0.42	2.40±0.08
**AF**	ND	0.21±0.01	0.23±0.01

### 3.2. In Vitro Antioxidant Assays

Since it is now recognized that there is no single test to evaluate antioxidant activities of the compounds with wide spectra of structures, modes of action, and physical and chemical properties, several different assays were employed as a part of our investigation. Of these, including DPPH· scavenging, ABTS^+^·scavenging, ·OH scavenging, ·O_2_^−^ scavenging, Cu^2+^-chelating and FRAP, anti-LP assays were most commonly used for the evaluation of antioxidant activities.

#### 3.2.1. DPPH• Scavenging Activity Assay

DPPH• is widely used for screening of medicinal plants to investigate their antioxidant potential. The principle of this antioxidant assay is the capability to diminish the color of DPPH•, an ethanol solution of stable free radical, in the presence of antioxidants. The deep purple color of DPPH• is due to the presence of an odd electron in it [26]. When an electron is donated by an antioxidant compound to DPPH•, the DPPH• is decolorized; this can easily be quantified by noting the change in absorbance at 515 nm. Unlike laboratory-generated free radicals such as the ·OH and ·O_2_^−^, DPPH• has the advantage of being unaffected by certain side reactions, such as metal ion chelating and enzyme inhibition [27].

**Figure 3:**
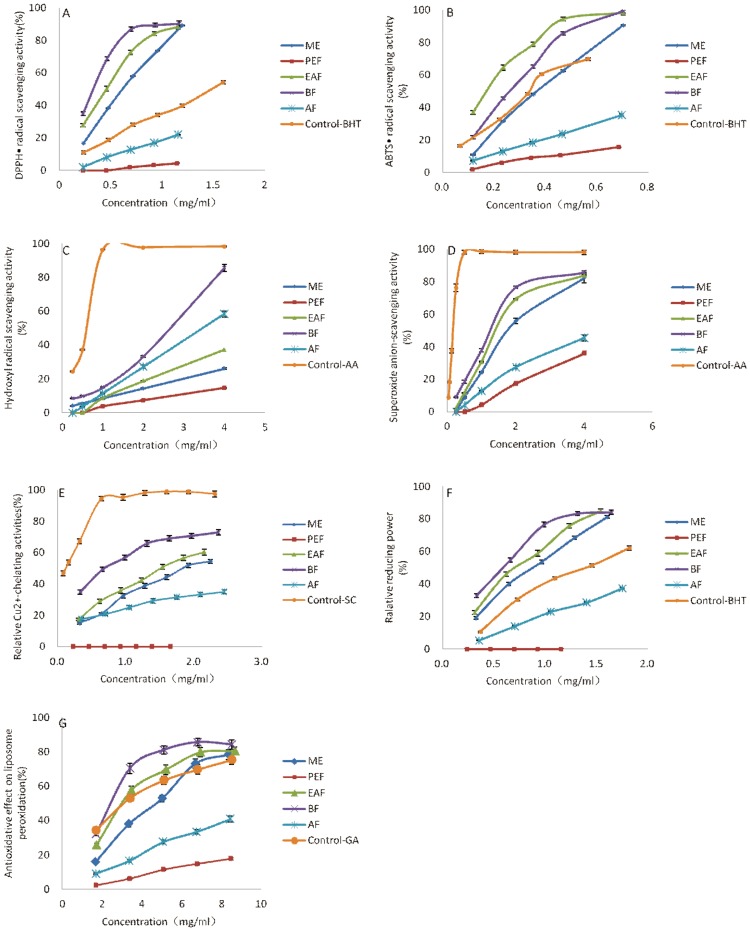
Antioxidant activities of ME and its four fractions of CK by seven methods. A. DPPH· scavenging activity assay, B. ABTS^+^· scavenging activity assay, C. ·OH scavenging activity assay, D. ·O_2_^−^ scavenging activity assay, E. Cu^2+^-chelating activity assay, F. FRAP, G. Antioxidant effect on LP.

Different fractions and ME from CK for DPPH• showed considerable scavenging activities in figure 3(A) and Table 3. BF revealed the outstanding scavenging activity, which was equivalent to quadruple amount of BHT, the control. Besides PEF, all the samples exhibited marked DPPH• scavenging activity in a concentration-dependent manner. The scavenging activities of BF, EAF, and ME above 0.234 mg/mL were stronger than the activity of BHT at the same concentration. According to the IC_50_ value of the samples and BHT, the order of their activities was BF > EAF > ME> BHT > AF > PEF. The results were found to be statistically significant (P<0.01) at IC_50_.

Zheng [28] had published his thesis in the year 2012; his thesis had also noted the antioxidant property of CK extractive fractions (BF and EAF) with respect to DPPH• scavenging test. In his research, BF (80%) had more marked DPPH• scavenging activity compared with EAF (40%) in the concentration of 0.1 mg/mL. This conclusion was similar to our result. Ning [6] detected antioxidant activity in aqueous extract of seven Callicarpa species using the method of DPPH• scavenging test; IC_50_ of CK was 0.85 mg/mL and all of the Callicarpa species ranged from 0.24 to 1.13 mg/mL. Since the amount of species in the two tests was different, the IC_50_ in the two tests can't be compared with each other. Lin [29] found that oils from *C. formosana* Rolfe at the concentration of 65.5 μg/mL displayed an ability to scavenge 50% DPPH• and showed a concentration-effect relationship; so we can infer that the oils from CK contained antioxidant compounds. Forsythiaside B, poliumoside, and acteoside are the main components in CK; the DPPH• scavenging activities of three PG and two control decreased in the following order: forsythiaside B =  poliumoside > acteoside > tocopherol > BHT [4]. We can infer tentatively that three PG are the main antioxidant compounds in CK.

#### 3.2.2. ABTS^+^• Scavenging Activity Assay

The ABTS^+^• scavenging activity assay is an indirect method that simply measures the activity of the ABTS^+^• to abstract an hydrogen atom or an electron from the compounds under study [30]. The simplicity of the method has made it very popular and suitable for assessing the ability of compounds to act as hydrogen/electron donors and to evaluate their antioxidant activity. ABTS^+^•, a cation free radical soluble in both water and organic media, is produced by reacting ABTS solution with potassium persulfate [31].

The differences of the ABTS^+^• scavenging activities of each sample and the control, BHT are recorded in Table 4 and figure 3(C). The ABTS^+^• scavenging activities of ME and its fractions increased with the increase of concentrations and decreased in the following order: EAF > BF > BHT > ME > AF > PEF (p<0.05). ABTS^+^• scavenging activity patterns may vary somewhat from those of DPPH• scavenging activity. The ABTS^+^• scavenging activity of BF above 0.117 mg/mL was significantly lower than that of EAF at the same concentration. some researches [32] has reported that the high molecular weight phenolics (tannins) had more ability to quench ABTS^+^• and their effectiveness depended on the molecular weight, the number of aromatic rings, and nature of hydroxyl group's substitution than the other specific functional groups in the high molecular weight phenolics. ABTS^+^• scavenging activity of EAF might be due to the presence of a greater number of high molecular weight phenolics compared to BF.

**Table 4:** IC_50_ of different extracts of CK for various antioxidant systems.

Sample	DPPH• radical scavenging activity assay	ABTS^+^• radical scavenging activity assay	Cu^2+^-Chelating activity Assay	FRAP
**ME**	0.62±0.018^d^	0.37±0.013^c^	1.85±0.084^a^	0.88±0.021^c^
**PEF**	13.54±0.480^a^	1.10±0.034^a^	ND	ND
**EAF**	0.46±0.017^e^	0.17±0.0061^f^	1.53±0.043^b^	0.71±0.019^d^
**BF**	0.32±0.007^f^	0.25±0.0074^e^	0.68±0.012^c^	0.55±0.008^e^
**AF**	3.92±0.130^b^	1.01±0.028^b^	ND	2.13±0.075^a^
**Control**	1.30±0.034^c^	0.33±0.016^d^	0.12±0.002^d^	1.38±0.051^b^

Each value in the table is represented as Mean ± SD (n = 3). Means not sharing the same letter are significantly different at P<0.05 probability level in each column.

In Singh's report [26],IC_50_ of ABTS^+^• scavenging activities in BHT, ethanol, acetone, and aqueous from *Callicarpa Macrophyla* were 0.0233±0.02, 0.0343±0.04, 0.0417±0.04, and 0.0429±0.04 mg/mL, respectively. Once again, BHT has proven to be comparatively more active in scavenging ABTS^+^•. Base on Singh's and our experiments, we can infer that it was possible to extract EAF primarily with ethanol rather than acetone.

Because DPPH• and ABTS^+^• don't exist in vivo, our present study further investigated the scavenging activities of CK against •OH, and •O_2_^−^ using two classical methods.

#### 3.2.3 •OH Scavenging Activity Assay

The •OH radical has a short half-life and is the most reactive and damaging ROS. It causes oxidative damage to DNA, lipids, proteins, and amino acids. •OH is known to be capable of abstracting hydrogen atoms from membranes and it brings about peroxidation reactions of lipids. It is generated in the human body via the Fenton reaction. •OH scavenging activity of CK is directly related to its antioxidant activity.

Figure 3(C) and Table 5 show the •OH scavenging activity at different concentration of the samples of CK along with the control, AA. The •OH scavenging activities of all samples were significantly lower against AA, which possess strong •OH scavenging activity. When the concentration of the samples was more than 1 mg/mL, all the samples showed dose-dependent inhibition of •OH. With regard to the •OH scavenging activity at 4 mg/mL, BF (85.47±2.10%) at 4 mg/mL was a considerably more effective (p<0.05) •OH scavenger compared to AF (58.36±1.70%), EAF (37.26±0.14%), ME (26.12±0.45%), and PEF (14.64±0.18%). Different from the above two free radical scavenging experiments, •OH scavenging activity of EAF was weaker than that of AF; that could be because the reaction ran in the polar medium, in which the EAF had a low dissolubility.

**Table 5:** •OH scavenging activity of different extracts of CK.

Sample	•OH scavenging activity, %				
	Concentration(mg/mL)				
	0.25	0.5	1	2	4
**ME**	3.98±0.12	5.45±0.18	8.46±0.38	14.33±0.35	26.12±0.45^b^
**PEF**	ND	ND	3.85±0.08	7.47±0.14	14.64±0.18^a^
**EAF**	ND	ND	8.73±0.14	18.70±0.17	37.26±0.14^c^
**BF**	8.44±0.24	9.95±0.20	15.01±0.33	33.28±0.38	85.47±2.10^e^
**AF**	ND	3.76±0.10	11.55±0.32	27.17±0.97	58.36±1.70^d^
**Control**	24.34±0.19	37.13±0.26	96.32±0.21	97.62±0.22	98.18±0.26^f^

Each value in the table is represented as Mean ± SD (n = 3). Means not sharing the same letter are significantly different at P<0.05 probability level in each column.

The result of Zheng's [28] •OH scavenging activity assay of BF and EAF of CK showed that •OH scavenging activity of EAF (29%) was slightly higher than BF(25%), which was very different from our conclusion. In Zheng's experiment, the reaction ran in the ethanol water mixture, in which EAF had a high dissolubility. In this system, EAF reflected a more strong •OH scavenging activity.

#### 3.2.4. •O_2_^−^ Scavenging Activity Assay

Besides •OH radical, •O_2_^−^ is also regarded as one important form of ROS in the living cell. Although •O_2_^−^ is a relatively weak oxidant, it can directly attack DNA or lipid and indirectly initiate lipid peroxidation, and form stronger reactive oxidative species such as single oxygen and hydroxyl radicals, which are able to kill cells, inactivate enzymes, and degrade polysaccharides, DNA, lipids, and possibly other susceptible substances of cells [33]. Thus, •O_2_^−^ is involved in many serious diseases, including neurodegenerative and cardiovascular disorders [34].

Figure 3(D) shows that all five samples of CK exhibited concentration-dependent •O_2_^−^ scavenging activity which was lower than that of the control, AA (P<0.05), significantly. BF (76.54±0.74%) possessed the most potent •O_2_^−^ scavenging activity followed by EAF (69.37±0.25%), ME(55.81±1.70%), AF(27.41±1.10%), and PEF(17.35±0.24%) at 2.0 mg/mL, as shown in Table 6. BF was still the most active in superoxide radical scavenging test.

**Table 6:** •O_2_^−^ scavenging activity of different extracts of CK.

Sample	•O_2_^−^ scavenging activity, %				
	Concentration(mg/mL)				
	0.25	0.5	1	2	4
**ME**	ND	8.78±0.21	24.41±0.29	55.81±1.70^c^	82.42±3.10
**PEF**	ND	ND	4.17±0.140	17.35±0.24^a^	35.94±0.26
**EAF**	1.92±0.07	11.55±0.28	30.80±0.37	69.37±0.25^d^	83.92±0.36
**BF**	8.78±0.15	18.45±0.89	37.88±0.87	76.54±0.74^e^	85.77±0.79
**AF**	ND	4.29±0.08	12.64±0.34	27.41±1.10^b^	45.41±1.80
**Control**	76.24±2.3	98.27±1.1	98.62±1.30	98.15±1.10^f^	98.28±1.30

Each value in the table is represented as Mean ± SD (n = 3). Means not sharing the same letter are significantly different at P<0.05 probability level in each column.

#### 3.2.5. Cu^2+^-Chelating Activity Assay

Transition metals, such as Fe and Cu, react very quickly with peroxides by acting as one-electron donors to form alkoxyl radicals. Therefore, the chelation of transition metal ions by antioxidant would retard the oxidation process [35], [36]. The research suggested that metal-chelating may be one of the mechanisms for scavenging •OH or •O_2_^−^. An earlier study showed that acidic and/or basic amino acids may play an important role in Fe^2+^ and Cu^2+^ chelation by peptides. So, the antioxidant activity can also be studied by the metal-chelating assay, which measures the protection ability that a sample offers against the oxidation activity catalyzed by transition metals, such as Fe^2+^and Cu^2+^ [37].

The data about the metal chelating activities of the samples and the control, SC, are listed in figure 3(E) and Table 4. The metal chelating activities of all the samples were significantly lower (p<0.05) than SC (0.118 mg/mL). In addition to AF, PEF, IC_50_ for the metal chelating activities varied significantly (p<0.05) among the samples and ranged from 0.68 to 1.85 mg/mL. IC_50_ for the metal chelating activities of AF and PEF could not reach the concentration at this test. It was obvious that BF was the most strong antioxidant in metal chelating activity (Table 4).

#### 3.2.6. Fe^3+^ Reducing Antioxidant Power

The reducing antioxidant power is often used as an indicator of electron donation and is one of the important antioxidant mechanisms [38]. The electron donor compounds are considered to be reducing agents and can eliminate the oxidized intermediates of the lipid peroxidation reactions; therefore they may be primary or secondary antioxidants [39].The FRAP test is a simple, reproducible, and reliable method to measure the ability of antioxidant activity [30]. According to this method, when the Fe^3+^/ferricyanide complex is reduced to the ferrous form (Fe^2+^-TPTZ), the absorbance of the formation of Perl's Prussian Blue at 700 nm is developed. [40].

The results obtained, except for PEF, are given in Table 4. IC_50_ values of FRAP of other four samples were respectively (0.55±0.0082 mg/mL, BF), (0.71±0.019 mg/mL, EAF), (0.88±0.021 mg/mL, ME), and (2.13±0.075 mg/mL, AF), while that of the control, BHT, was (1.38±0.051 mg/mL). FRAP of four samples and BHT increased with increasing concentration significantly (P<0.05). In addition to PEF and AF, the other three samples exhibited higher activity than BHT at all concentrations.

Singh [26] also researched the FRAP among the different extracts of *Callicarpa Macrophyla*. Of the three extracts, ethanol extract exhibited the highest FRAP (OD: 0.454±0.001) at 1 mg/mL. This was following by acetone extract (OD: 0.421±0.002) and aqueous extract (OD: 0.14±0.002). *Callicarpa Macrophyla* and CK display considerably excellent FRAP as reducing agents.

#### 3.2.7. Antioxidant Effect on Lipid peroxidation

Lipid peroxidation can be inherited by •OH through attacking biomolecules, especially polyunsaturated fatty acids, which easily appear LP initiated by free radical chain reactions in biological systems. The best-known LP product is MDA and it has been used most widely as a biomarker in various studies associated with LP. Determination of MDA may be problematic because of its high reactivity and water solubility, and therefore it is necessary to generate stable derivatives. One of the most commonly used derivative is (TBA)_2_-MDA which can be determined using spectrophotometry [41]. Since the samples of CK showed foreseeable scavenging activity against ROS, we further selected the soybean lecithin as the oxidizable biomolecular target for the Fe^2+^/ascorbate method to investigate the LP-inhibiting effect of CK.

The anti-LP effects of five samples and the control, AA, on nonenzymatic peroxidation are summarized in Table 7 and figure 3(G). The highest activity was observed for BF of CK at 5.1 mg/mL and was more potent in inhibition of LP than other samples. The anti-LP affects of five samples and AA decreased in the order: BF > EAF > AA > ME > AF > PEF, which were (81.12±2.40%), (69.60±2.80%), (63.35±2.40%), (63.35±2.40%), (52.75±2.00%), (27.48±1.10%), (11.36±0.47%) at 5.1 mg/mL, respectively.

**Table 7:** Antioxidant effect on LP of different extracts of CK.

Sample	Antioxidant effect on lipidperoxidation, %				
	Concentration(mg/mL)				
	1.7	3.4	5.1	6.8	8.5
**ME**	15.77±0.45	37.90±1.60	52.75±2.00^c^	73.14±2.70	78.42±2.90
**PEF**	2.38±0.08	6.19±0.24	11.36±0.47^a^	14.73±0.53	17.66±0.64
**EAF**	25.73±1.1	57.51±2.50	69.60±2.80^e^	79.85±2.60	80.57±2.10
**BF**	32.13±0.97	70.34±2.90	81.12±2.40^f^	85.62±2.30	84.27±2.70
**AF**	8.95±0.37	16.52±0.71	27.48±1.10^b^	33.43±1.40	40.85±1.80
**Control**	34.26±1.5	52.99±1.9	63.35±2.40^d^	69.72±2.90	75.30±2.60

Each value in the table is represented as Mean ± SD (n = 3). Means not sharing the same letter are significantly different at P<0.05 probability level in each column.

Huang [42] has reported a research on LP of tissue homogenate of liver, kidney, heart and brain of rat. LP was inhibited markedly by the water extract of *Callicarpa cathayana H.T.* Chang. Another research [43] on anti-LP test was carried out on six Callicarpa species, and the anti-LP activities decreased in this order: *C. Kochiana* > *C. Japonica* > *C. Bodinieri* > *C. Macrophylla* >*C. Cathayana* > *C.giraldii*, and the most noteworthy was that *C.giraldii* was not able to show anti-LP activity. In addition, forsythiaside B, poliumoside, acteoside from *C. japonica Thunb. var. luxurians* Rehd. were mentioned as anti-LP reagents stronger than tocopherol and BHA; we can infer tentatively that three PG are main anti-LP compounds in CK [4].

#### 3.2.8. Antioxidant Activity of BF on CCl_4_-induced acute liver injury mice

Many antioxidant tests in vitro have proved that BF was the fraction of the strongest antioxidant activity in different extracts of CK, an antioxidant experiment in vivo was designed to verify that the conclusion was correct. CCl_4_-induced liver injury often leads to abnormal MDA concentration and SOD activity which are the most important indexes about antioxidant activities in CCl_4_-induced liver injury.

To evaluate the effect of BF pretreatment on CCl_4_-induced liver injury, MDA concentration and SOD activity was monitored. It was found that administering CCl_4_ increased the hepatic level of MDA by about 2.1-fold compared to the control animals (Table 8). This elevation was mitigated after administration of BF and berberine, BF inhibited the production of MDA in a obvious dose-effect manner. According to the amount of MDA, the order was Control ≈ Berberine ≈ BF (900 mg/kg) > BF (300 mg/kg) > BF (100 mg/kg) > CCl_4_.Compared to the CCl_4_ group, the level of SOD of liver increased significantly in BF and berberine groups (*p*<0.05). The order of their activities were Control > Berberine > BF (900 mg/kg) > BF (300 mg/kg) ≈ BF (100 mg/kg) > CCl_4_.

**Table 8:** Effect of BF on the level of hepatic MDA and SOD activity in mice treated with CCl_4_.

Groups	MDA	SOD Activity
	(nmoL/mg protein)	(U/mg protein)
**Control**	7.39±1.45^a^	265.630±18.375^a^
**CCl_4_**	15.65±1.89^d^	96.802±10.368^e^
**Berberine 40 mg/kg+CCl_4_**	8.12±1.83^a^	221.728±19.472^b^
**BF 100 mg/kg+CCl_4_**	13.64±1.98^c^	115.657±10.670^d^
**BF 300 mg/kg+CCl_4_**	11.81±1.63^b^	117.358±11.964^d^
**BF 900 mg/kg+CCl_4_**	8.25±1.76^a^	185.577±14.845^c^

Each value in the table is represented as Mean ± SD (n = 8). Means not sharing the same letter are significantly different at P<0.05 probability level in each column.

The results of the present study indicated that BF increased SOD activity and decreased MDA activity, consequently increased the antioxidant activities of liver. No one has used the antioxidant methods in vivo to study the Callicarpa species in terms of liver protection.

#### 3.2.9 Determination of Cytotoxicity/Protective Effect of HepG2 cells

The viability of HepG2 cells after treatment with various concentrations of BF (0.04–5 mg/mL) for 24 h was more than 47%, and the viability after treatment with BF (25 mg/mL) was too low (20.98±2.7%) to be chosen for the investigation of protective effect of BF on the cells (Table 9). Except for BF (0.04 mg/mL), all BF and VC showed a protective effect on TBHP-induced HepG2 cells (Table 9). The cell viability was decreased by concentrations of BF under a dose-effect relationship. BF (5 mg/mL) group was only slightly higher than BF (2.5 mg/mL), which may be because the cytotoxicity of BF (5 mg/mL) on HepG2 cells was far higher than that of BF (1 mg/mL).

**Table 9:** Cytotoxicity and protective effect of BF on HepG2 cells.

Groups	Cell viability(%)	
	Cytotoxicity of BF on HepG2 cells	Protective effect of BF on TBHP-induced HepG2 cells
**Contol(cell only)**	100±2.931	-
**Control(cell+0.5 mM TBHP)**	-	46.49±1.3^a^
**VC(2.5 μg/mL)**	-	85.67±0.5^c^
**BF(0.04 mg/mL)**	94.40±1.6	44.86±0.4^a^
**BF(0.2 mg/mL)**	89.40±1.7	80.51±4.7^b^
**BF(1 mg/mL)**	84.60±3.7	89.42±1.4^d^
**BF(5 mg/mL)**	47.02±1.0	91.30±0.3^e^
**BF(25 mg/mL)**	20.98±2.7	-^a^

Each value in the table is represented as Mean ± SD (n = 5). Means not sharing the same letter are significantly different at P<0.05 probability level in each column. a. the viability after treatment with BF (25 mg/mL) was too low to be chosen for the investigation of protective effect of BF on the cells.

A few researchers [44], [45]have worked on the cell experiments in terms of antioxidant, acteoside isolated from the leaves of *Callicarpa dichotomahas* significant enhanced neuroprotective activity against glutamate-induced neurotoxicity in primary cultured rat cortical cells and ten phenylethanoid glycosides including acteoside isolated from the leaves and twigs of *Callicarpa dichotoma* significantly attenuated glutamate-induced neurotoxicity.

### 3.3. Correlations of Antioxidant Activities and TPC, TFC of CK

The relationship between antioxidant activities and TPC, TFC of CK was shown in Table 10. There were significant positive correlations between DPPH• scavenging activity (0.840a), ABTS^+^• scavenging activity (0.905a), •O_2_^−^ scavenging activity (0.974b), Cu^2+^-chelating activity (0.998a), antioxidant effect on anti-LP (0.987b) and TPC in various fractions of extract while non-significant correlation was observed between •OH scavenging activity (0.687), FRAP (0.885) and TPC. Non-significant correlations were present between various antioxidant activities and TFC in this study. Ning [6] has reported that the correlations between TPC, TFC and DPPH• scavenging activity of seven Callicarpa species were 0.982 4b, 0.991 5b, respectively. Our data were similar for TPC but not TFC, because TFC in CK was rather lower than that in other Callicarpa species.

**Table 10:** Pearson correlation coefficients between antioxidant activities and TPC, TFC.

Assays	Pearson correlation coefficient (r)	
	TPC	TFC
**DPPH• scavenging activity**	0.840^a^	0.338
**ABTS^+^• scavenging activity**	0.905^a^	0.211
**•OH scavenging activity**	0.687	0.631
**•O_2_^−^ scavenging activity**	0.974^b^	0.393
**FRAP**	0.885	0.381
**Cu^2+^-chelating activity**	0.998^a^	0.614
**Antioxidant effect on anti-LP**	0.987^b^	0.439

a. p<0.05;

b. p<0.01.

### 3.4. Rapid Screening of Antioxidants from CK by HPLC-UV

**Figure 4:**
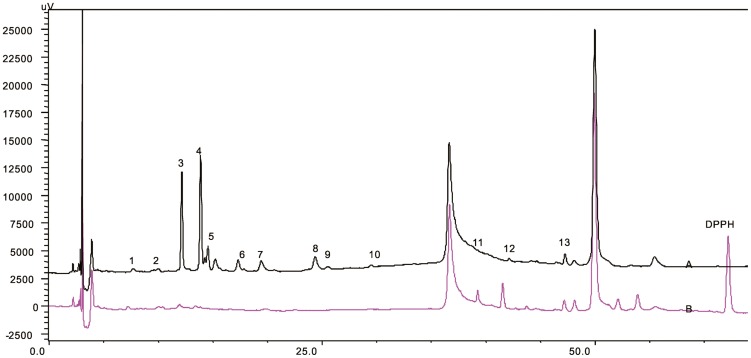
Chromatograms of ME of CK before and after teaction with DPPH•. A. Chromatograms of ME of CK before reaction with DPPH·; B. Chromatograms of ME of CK after reaction with DPPH· at 254 nm.

The results obtained are given in figure 4 and Table 11. The peak of DPPH appeared in Chromatogram B, which pointed out that DPPH was excess in the reaction with ME and ensured that antioxidants in ME can be reacted, no matter how active their antioxidant abilities. Compound 1–9 belonged to the PG and had excellent antioxidant ability, because this type of compounds possess caffeoyl, acetyl moiety, and phenolic hydroxyl group which always play the role of reducing group. A lot of researches [46] proved PG owned antioxidant ability, which was consistent with our study. Apart from nine PG, compound 13 (Rhamnazin) also showed good antioxidant ability, in accordance with their reports. But it was strange that after reaction with DPPH•, the peak area of compound 11,12 increased too much; it might be because some unknown compound has oxidized these compounds after reaction with DPPH•; so compound 11,12 cannot be identified as antioxidant temporarily. Compound 10 also cannot be identified as antioxidant because it was not detected before or after reaction with DPPH•.

**Table 11:** RBA of 13 compounds from ME of CK to DPPH•.

Peak	Compound	RBA (*n* = 3)	Antioxidant
**1**	β-OH-forsythoside B	100%±0^a^	Y
**2**	β-OH-poliumoside	100%±0^a^	Y
**3**	Forsythoside B	96.51%±1.4%	Y
**4**	Poliumoside	89.85%±1.1%	Y
**5**	Acteoside	87.10%±1.1%	Y
**6**	Alyssonoside	95.50%±1.3%	Y
**7**	2′-forsythoside B	75.00%±0.8%	Y
**8**	Brandioside	93.02%±1.1%	Y
**9**	2′-acetylacteoside	100%±0^a^	Y
**10**	2′,7″- diacetoxylacteoside	-^b^	N
**11**	3,4-dimethoxy,β-OH- 2′- acetyacteoside	-^c^	N
**12**	Acacetin-7-*O*-diglucuronide	-^c^	N
**13**	Rhamnazin	100%±0^a^	Y

a. Relative binding affinity 100% was thought to be reacted completely because it was not detected in experiment group;

b. Compound 10 can't detected before or after reaction with DPPH• and identified as antioxidants;

c. The peak area of compound 11, 12 increased too much and identified as antioxidants temporarily.

## Conclusions

Abundant polyphenolic content of BF in CK reflected antioxidant properties by some antioxidant methods including DPPH• scavenging, ABTS^+^• scavenging, •O_2_^−^ scavenging, Cu^2+^-chelating, and antioxidant effect on anti-LP. The antioxidant compounds consisted of nine PG (forsythiaside B, poliumoside, acteoside, alyssonoside, brandioside and their derivatives) and one flavone (rhamnazin) at least. Our research was beneficial to develop CK as a safe and efficient antioxidant. The test dosage of BF of CK in our each experiment proved that BF was an efficient antioxidant, and the next step is to verify a concentration to distinguish BF acting as antioxidant or pro-oxidant.
